# Effects of FAP^+^ cancer-associated fibroblasts on anti-PD-1 immunotherapy and CD4^+^ T cell polarization in gastric cancer

**DOI:** 10.20517/cdr.2025.97

**Published:** 2025-07-29

**Authors:** Jing Wu, Peng-Fei Zhang, Yu Zeng, Ya-Nan Hai, Kun-Ming Zhang, Shu Dong, Ji-Chong Xu, Lan-Lin Zhang, Zhi-Xiong Wu, Hong Jiang

**Affiliations:** ^1^Department of Cancer Center, Tongji Hospital, Tongji University School of Medicine, Shanghai 200065, China.; ^2^Department of Medical Oncology, Zhongshan Hospital, Fudan University, Shanghai 200032, China.; ^3^Department of Medical Oncology, Shanghai Geriatric Medical Center, Shanghai 201104, China.; ^4^Department of Pathology, Tongji Hospital, Tongji University School of Medicine, Shanghai 200065, China.; ^5^Department of Oncology, Shanghai East Hospital, Tongji University School of Medicine, Shanghai 200120, China.; ^6^Department of Oncology, People’s Hospital of Longhua, Shenzhen 518109, Guangdong, China.; ^7^Department of Integrative Oncology, Fudan University Shanghai Cancer Center, Shanghai 200032, China.; ^8^Department of Interventional Radiology, Tongji Hospital, Tongji University School of Medicine, Shanghai 200065, China.; ^#^Authors contributed equally.

**Keywords:** Cancer-associated fibroblasts, immune evasion, anti-PD-1 therapy, naive CD4^+^ T cell differentiation

## Abstract

**Aim:** The immune evasion mechanisms of gastric cancer are complex, involving various cellular dysfunctions within the tumor microenvironment. Recently, there has been growing interest in how cancer-associated fibroblasts (CAFs) contribute to tumor immune evasion. However, the precise molecular pathways through which CAFs drive immune escape in the context of gastric cancer are not yet fully elucidated.

**Methods:** The abundance of FAP^+^CAFs in gastric cancer tissues was assessed by immunohistochemistry (IHC), and its correlation with tumor sensitivity to PD-1 monoclonal antibody therapy was analyzed. To study the effect of FAP^+^CAFs on naive CD4^+^ T cell differentiation, co-culture experiments were conducted. The underlying molecular mechanisms were further investigated through western blotting and *in vivo* animal experiments.

**Results:** FAP^+^CAFs were significantly increased in gastric cancer tissues resistant to PD-1 monoclonal antibody, and a positive correlation was found with Th2 cells. Additionally, the expression and secretion of IL-31 in FAP^+^CAFs cells were elevated. Mechanistically, IL-31 interacts with the IL-31R expressed on naive CD4^+^ T cells, leading to the activation of the STAT6 signaling pathway. This cascade facilitates the differentiation of naive CD4^+^ T cells into Th2 cells, thereby contributing to resistance against anti-PD-1 therapy in gastric cancer.

**Conclusion:** FAP^+^CAFs may reduce sensitivity to anti-PD-1 therapy in gastric cancer by promoting Th2 polarization of naive CD4^+^ T cells via the IL-31/STAT6 signaling pathway. Targeting this axis could offer a potential strategy to improve immunotherapy outcomes, although further validation is required.

## INTRODUCTION

As a significant global health concern, gastric cancer ranks fifth in incidence and third in mortality among all cancer types^[1]^. In recent years, immune checkpoint inhibitors, particularly PD-1/PD-L1 inhibitors, have shown promise in improving patient outcomes^[2,3]^. However, despite these advances, a significant proportion of patients either exhibit primary resistance to anti-PD-1 therapy or develop secondary resistance after initial responsiveness^[4-6]^. The mechanisms underlying this resistance are complex and involve abnormal communication among cancer cells, immune cells, and stromal components within the tumor microenvironment (TME)^[4,7-9]^. Thus, understanding the molecular pathways that drive immune evasion is critical for overcoming resistance and enhancing the therapeutic performance of immune checkpoint blockade in gastric cancer.

Cancer-associated fibroblasts (CAFs) are the most abundant stromal cells within the TME and are known to play a crucial role in tumor progression and immune evasion across various malignant cancers^[10-12]^. CAFs exert their function through multiple mechanisms, including the secretion of cytokines and exosomes, which collectively affect tumor energy metabolism, enhance angiogenesis, disrupt immune cell functionality, and remodel the extracellular matrix. These processes ultimately facilitate tumor advancement and immune evasion^[13,14]^. For example, CAFs are independent prognostic factors for patient survival and closely associated with poor prognosis in lung cancer^[15]^. In gastric cancer, CAFs promote immune escape by inducing NK cell ferroptosis^[16]^. Additionally, POSTN^+^CAFs are linked to low abundance and functional exhaustion of T cells in advanced lung cancer tumors^[17]^.

Recent studies suggest that CAFs may exhibit significant heterogeneity, with specific subsets of CAFs playing distinct roles in immune modulation. Among these, FAP^+^CAFs have been implicated in promoting tumor progression and modulating the immune response^[18,19]^. However, their role in the context of immune checkpoint blockade resistance in gastric cancer has yet to be fully elucidated. This gap in knowledge underscores the importance of investigating the molecular mechanisms by which FAP^+^CAFs influence immune escape and reduced responsiveness to PD-1 therapy.

In this study, we found high expression of FAP in CAFs from gastric cancer tissues resistant to anti-PD-1. Mechanistically, CAFs overexpressing FAP upregulate IL-31 secretion, thereby enhancing the activity of the IL-31R/STAT6 axis, promoting the polarization of naive CD4^+^ T cells toward a Th2 phenotype. Ultimately, this leads to resistance of gastric cancer to anti-PD-1. This study reveals a novel mechanism driving immune evasion in gastric cancer, proposes FAP^+^CAFs as a potential biomarker for predicting anti-PD-1 therapy resistance, and suggests that targeting the IL-31/STAT6 axis could offer new therapeutic strategies to enhance immunotherapy efficacy.

## METHODS

### Tissues

Gastric cancer tissues were used for immunohistochemical staining or CAF isolation. Pathological confirmation was provided by pathologists. Clinical and pathological information was collected from January 1, 2020, to December 31, 2023. The study was approved by the Ethics Committee of our Hospital and informed consent was obtained from each patient.

### Immunohistochemistry

Immunohistochemistry (IHC) staining was conducted following protocols established in our earlier work. Formalin-fixed, paraffin-embedded tissue sections (4 μm thick) were used to evaluate FAP expression through an immunoperoxidase-based detection method. Staining intensity and positive area were quantified using the MetaMorph Imaging System (version 3.0; Universal Imaging Corp, Buckinghamshire, UK).

### Correlation analysis between FAP^+^CAFs in tumor tissues and CD4^+^ or CD8^+^ T cells in blood

The infiltration density of FAP^+^CAFs in tumor tissues was assessed by IHC, with five fields randomly selected for quantification. The counts of CD4^+^ T and CD8^+^ T cells in blood were obtained from the immune cell subset reports issued by the Department of Laboratory Medicine. Then, a correlation analysis between the infiltration density of FAP^+^CAFs in tumor tissues and the levels of peripheral CD4^+^ or CD8^+^ T cells was performed. FAP^+^CAFs were systematically counted in tumor sections, and patients were dichotomized into high *vs.* low FAP^+^CAFs groups based on median counts.

### Cell culture

Human gastric cancer cell line AGS and mouse gastric cancer cell line MFC were purchased from the Cell Bank of Chinese Academy of Sciences and cultured in DMEM supplemented with 10% FBS and 1×penicillin-streptomycin. All co-culture experiments in this study were conducted using a non-contact trans-well system.

### CAFs isolation

The method for CAFs isolation was adapted from previously published literature with minor modifications. The detailed procedure is provided in the Supplementary Experimental Methods.

### Flow cytometry

The cells to be analyzed were incubated with FITC-labeled anti-CCR4 (Chemical book; CB76379525) or anti-Ki-67 (Invitrogen; 11-5699-42) antibody. After washing, labeled cells were acquired on a CytoFLEX flow cytometer (Beckman Coulter, Brea, CA, USA), and data analysis was performed with FlowJo software (v10.8.1).

Naive CD4^+^ T cells were negatively selected from PBMCs using a commercial isolation kit (Human Naive CD4^+^ T Cell Isolation Kit, Miltenyi Biotec, 130-094-131) following the manufacturer’s standardized protocol to ensure cell purity.

### Detection of secretory proteins and serum proteins

Plasma and cell culture supernatant levels of IL-31 (Elabscience, E-EL-H5469), IFN-γ (Elabscience, E-EL-H0108), perforin (Elabscience, E-EL-H1123), and IL-4 (Elabscience, E-EL-H0101) were measured by ELISA kits according to the manufacturer’s instructions. The patients in each panel were divided into IL-31^high^ and IL-31^low^ groups using median cutoff values.

### Western blot

The western blotting procedure was adapted from our previously published study. In brief, cell lysates were centrifuged at 13,000 ×*g* for 15 min at 4 °C, and the resulting supernatant was transferred into fresh microcentrifuge tubes. Protein concentrations were assessed using a BCA assay kit (Pierce, Rockford, IL, USA). Equal amounts of total protein were loaded onto 10% or 12.5% SDS–polyacrylamide gels for electrophoretic separation, followed by transfer to nitrocellulose membranes. Membranes were blocked with 5% nonfat milk and incubated with primary antibodies overnight at 4 °C. After washing, HRP-linked secondary antibodies were applied for 2 h at room temperature. Signals were detected using an enhanced chemiluminescence (ECL) system.

### Animal model of 615 mice

Four-week-old male 615 mice were subcutaneously injected with 1 × 10^6^ MFC murine gastric cancer cells in the right posterior axillary region. When tumor volumes reached approximately 50 mm^3^, the mice were randomized into four treatment groups: PD-1 mAb (Generously provided by BeiGene, Ltd. Intraperitoneal injection at 125 μg/dose, twice weekly), IL-31 (Sinobiological, 51070-M08H. Recombinant murine IL-31 group subcutaneous injection at 1 μg/dose, twice weekly), PD-1 mAb + IL-31, and IgG control. The experimental endpoint was defined as humane euthanasia by cervical dislocation.

### Preparation of humanized mice and anti-PD-1 therapy

NOG-EXL mice were obtained from Beijing Vital River Laboratory and housed under sterile conditions at the Experimental Animal Center. Newborn NOG-EXL mice underwent a 2Gy radiation treatment, followed by intravenous infusion of 5 × 10^4^ CD34^+^ human hematopoietic stem cells (HSCs) via the tail vein. At 7-8 weeks post-transplantation, peripheral blood samples were obtained and assessed via flow cytometry to assess human immune cell reconstitution. If the proportion of human CD45^+^ cells was greater than 45%, the huHSC-NOG-EXL mice were considered successfully established. Tumor transplantation experiments were conducted between weeks 8-11. Approximately AGS cells 3 × 10^6^ tumor cells and 3 × 10^6^ CAFs were suspended in 100 μL DMEM medium and implanted subcutaneously into the right flank of huHSC-NOG-EXL mice. Once the tumors reached approximately 50 mm^3^, anti-PD-1 antibody (BeiGene, Ltd., intraperitoneal injection at 125 μg/dose, twice weekly), anti-IL-31 antibody (Sinobiological, 11557-MM03, intraperitoneal injection at 125 μg/dose, twice weekly), or control IgG were administered intraperitoneally three times a week. Tumor dimensions were recorded every two days.

### Statistical analysis

Data analysis was conducted with SPSS 23.0 (Chicago, IL). Data are expressed as mean ± standard deviation. Student’s *t*-test was used to compare differences between two groups, while categorical variables were compared using the chi-square test or Fisher’s exact test. Spearman correlation analysis was used to assess the correlation between the expression levels of circulating molecules. The Kaplan-Meier estimator and log-rank test were applied to assess prognostic differences in gastric cancer patients. Chi-square tests were applied when expected frequencies in contingency tables were ≥ 5. Fisher’s exact tests were used for small sample sizes or expected frequencies < 5. The bar graphs were generated using GraphPad Prism Version 10.1.2, and the figure panels were assembled using Adobe Illustrator 2024. All *P*-values were two-sided, and *P* < 0.05 was considered statistically significant.

## RESULTS

### FAP^+^CAFs subpopulation in the tumor tissues of gastric cancer associated with anti-PD-1 therapy resistance and worse prognosis

**Figure 1:**
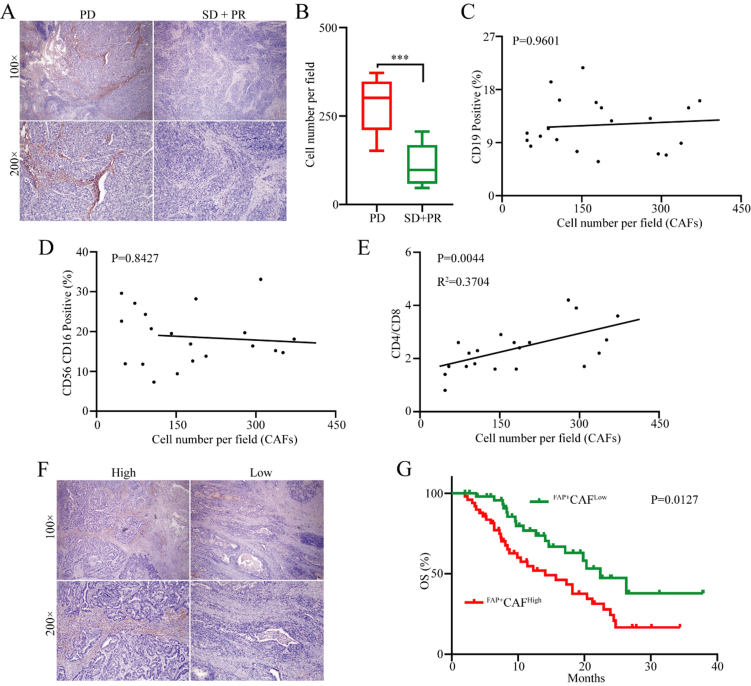
The abundance of FAP^+^CAFs in tumor tissues is associated with resistance to anti-PD-1 and poor prognosis in GC. (A and B) Immunohistochemical analysis of the relationship between the abundance of FAP^+^CAFs in gastric cancer tissues from patients receiving frontline immunotherapy and sensitivity to anti-PD-1 (PD: 8 patients; SD + PR: 12 patients); (C) The relationship between the abundance of FAP^+^CAFs in gastric cancer tissues and the proportion of B cell subpopulations in peripheral blood (CD19/CD45); (D) The relationship between the abundance of FAP^+^CAFs in gastric cancer tissues and the proportion of NK cell subpopulations in peripheral blood (CD16^+^CD56/CD45); (E) The relationship between the abundance of FAP^+^CAFs in gastric cancer tissues and the CD4/CD8 ratio in peripheral blood; (F) Immunohistochemical analysis of the abundance of FAP^+^CAFs in gastric cancer tissues from GC patients; (G) The relationship between the abundance of FAP^+^CAFs in gastric cancer tissues and patient prognosis. ^***^P < 0.001; bars: means ± standard deviation. CAFs: Cancer-associated fibroblasts; GC: gastric cancer.

Although previous studies have found an association between FAP^+^CAFs and tumor progression as well as immune evasion^[18,19]^, whether FAP^+^CAFs play a significant role in immune evasion specifically in gastric cancer remains unclear. Given the crucial role of immune evasion in gastric cancer resistance to PD-1 monoclonal antibody therapy, we conducted immunohistochemical analysis of FAP^+^CAF expression levels in tumor tissues from 20 gastric cancer patients who received first-line anti-PD-1 therapy [Supplementary Table 1]. The results indicated that the proportions of FAP^+^CAFs were significantly increased in patients with progressive disease (PD) than in those with partial response (PR) and stable disease (SD) [Figure 1A and B]. To further clarify whether the abundance of FAP^+^CAFs is associated with the efficacy of antitumor immune therapy in gastric cancer patients, we performed a retrospective analysis of peripheral blood immune cell subsets from 20 patients. The results showed that the abundance of FAP^+^CAFs had no significant correlation with the proportions of B cells (CD19^+^) or NK cells (CD56^+^CD16^+^) [Figure 1C and D]. Interestingly, FAP^+^CAFs were positively correlated with the CD4/CD8 ratio [Figure 1E]. In order to further investigate the clinical significance of FAP^+^CAFs in gastric cancer, the infiltration of FAP^+^CAFs was measured in 100 (stage IV) gastric cancer tissues using IHC [Figure 1F and Supplementary Table 2]. Kaplan-Meier survival analysis showed that patients with high infiltration of FAP^+^CAFs showed shorter overall survival (OS) compared to those with lower levels of FAP^+^CAF infiltration [Figure 1G].

### FAP^+^CAFs promote Th2 polarization of naive CD4^+^ T cells via IL-31 secretion

**Figure 2:**
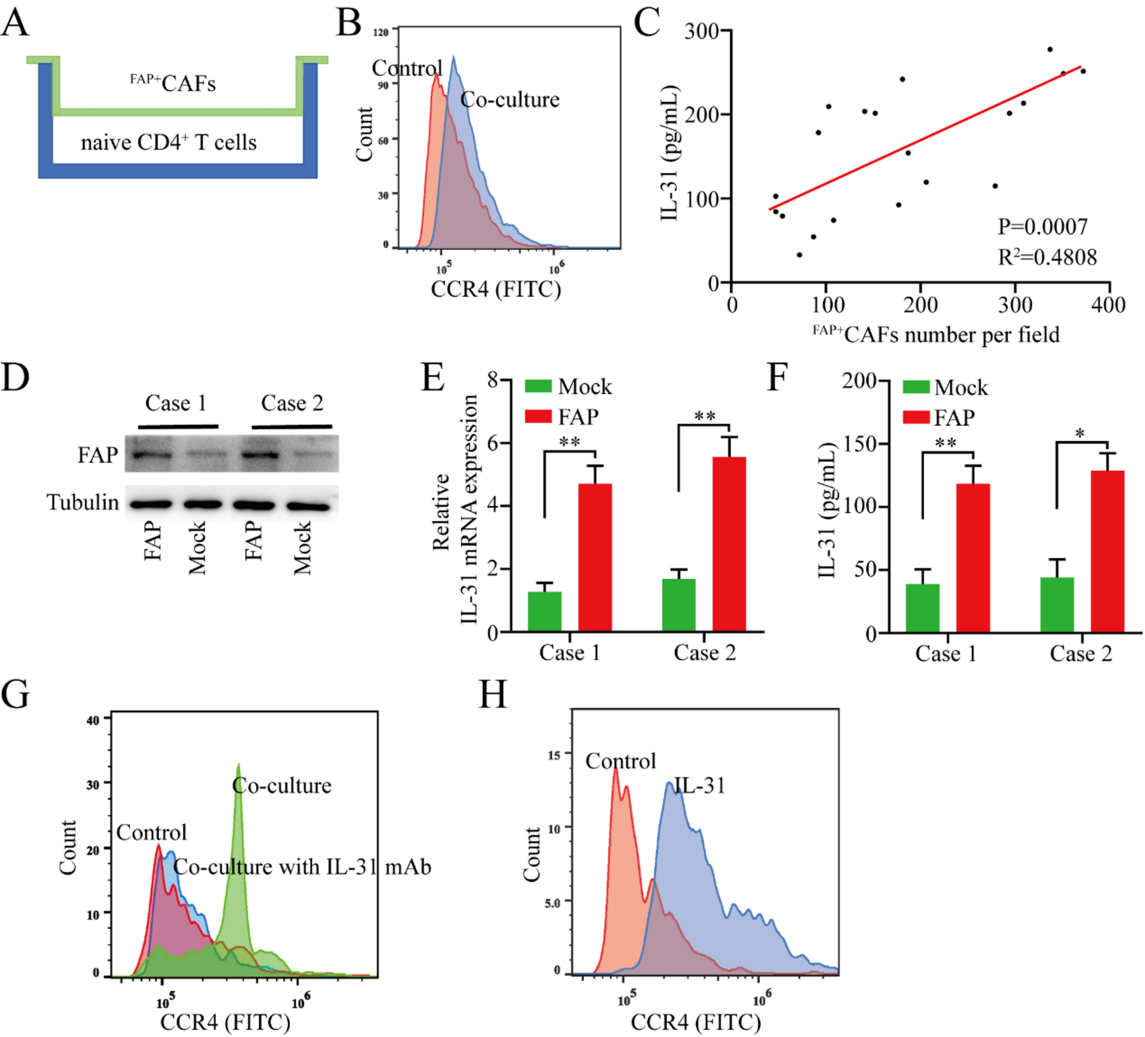
FAP^+^CAFs promote the polarization of naive CD4^+^ T cells toward Th2 by secreting IL-31. (A) Schematic of the co-culture model of naive CD4^+^ T cells and FAP^+^CAFs; (B) Flow cytometric analysis of CCR4 expression changes in naive CD4^+^ T cells after co-culture with FAP^+^CAFs; (C) Positive correlation between IL-31 expression levels in peripheral blood and the abundance of FAP^+^CAFs in tumor tissues; (D) To clarify the effect of FAP on IL-31 expression in CAFs, FAP expression was upregulated in isolated CAFs from gastric cancer tissues using lentiviral vectors; (E and F) Upregulation of FAP expression in CAFs was accompanied by increased IL-31 mRNA and protein expression (*n* = 3); (G) Flow cytometric analysis of whether the Th2 polarization (CCR4 expression) of naive CD4^+^ T cells mediated by FAP^+^CAFs can be blocked by IL-31 antibodies; (H) Flow cytometric analysis of the effect of rh-IL-31 on CCR4 expression in naive CD4^+^ T cells. ^*^P < 0.05; ^**^P < 0.01; bars: means ± standard deviation. CAFs: Cancer-associated fibroblasts.

Given that the abundance of FAP^+^CAFs in tumor tissues is positively correlated with the CD4/CD8 ratio in peripheral blood, and considering previous research indicating that Th1 and Th2 cells play markedly different roles in antitumor immunity - with Th1 cells primarily driving antitumor responses and Th2 cells facilitating immune evasion - we hypothesize that FAP^+^CAFs may promote the differentiation of CD4^+^ naive cells into Th2 cells through cytokine secretion. To test this hypothesis, naive CD4^+^ T cells isolated from six healthy donors were co-cultured with FAP^+^CAFs at a 1:1 ratio for 72 h [Figure 2A]. Compared to naive CD4^+^ T cells cultured alone, those co-cultured with FAP^+^CAFs demonstrated significantly increased CCR4 expression [Figure 2B].

Previous studies have found that IL-31 is overexpressed in dermal fibroblasts of patients with systemic sclerosis, which is characterized by fibrosis and autoimmune dysfunction. IL-31 directly promotes collagen production in dermal fibroblasts, thereby promoting fibrosis and Th2 polarization in systemic sclerosis^[20]^. Based on this, we speculate that FAP^+^CAFs may induce polarization of naive CD4^+^ T cells to the Th2 subtype by secreting IL-31. We detected IL-31 expression in the serum of the above 20 gastric cancer patients by ELISA, and the results of correlation analysis suggested that serum IL-31 expression levels were positively correlated with the abundance of FAP^+^CAFs in tumor tissue [Figure 2C]. To clarify the effect of FAP on IL-31 expression in CAFs cells, we isolated CAFs from two gastric cancer tissues and further upregulated FAP expression in CAFs using lentiviral vectors. The results showed that upregulation of FAP expression in CAFs was accompanied by increased IL-31 mRNA and protein expression [Figure 2D-F]. In addition, Th2 polarization in naive CD4^+^ T cells co-cultured with FAP^+^CAFs was reduced by IL-31 blocking antibodies [Figure 2G]. To further investigate whether the effect of FAP^+^CAFs on naive CD4^+^ T cells was mediated in an IL-31-dependent manner, naive CD4^+^ T cells were cultured with recombinant human IL-31 (rh-IL-31) for 72 h. The results showed that IL-31 effectively promoted the polarization of naive CD4^+^ T cells into the Th2 subtype [Figure 2H].

### IL-31 induces naive CD4^+^ T cells polarize into Th2 through the STAT6 axis

**Figure 3:**
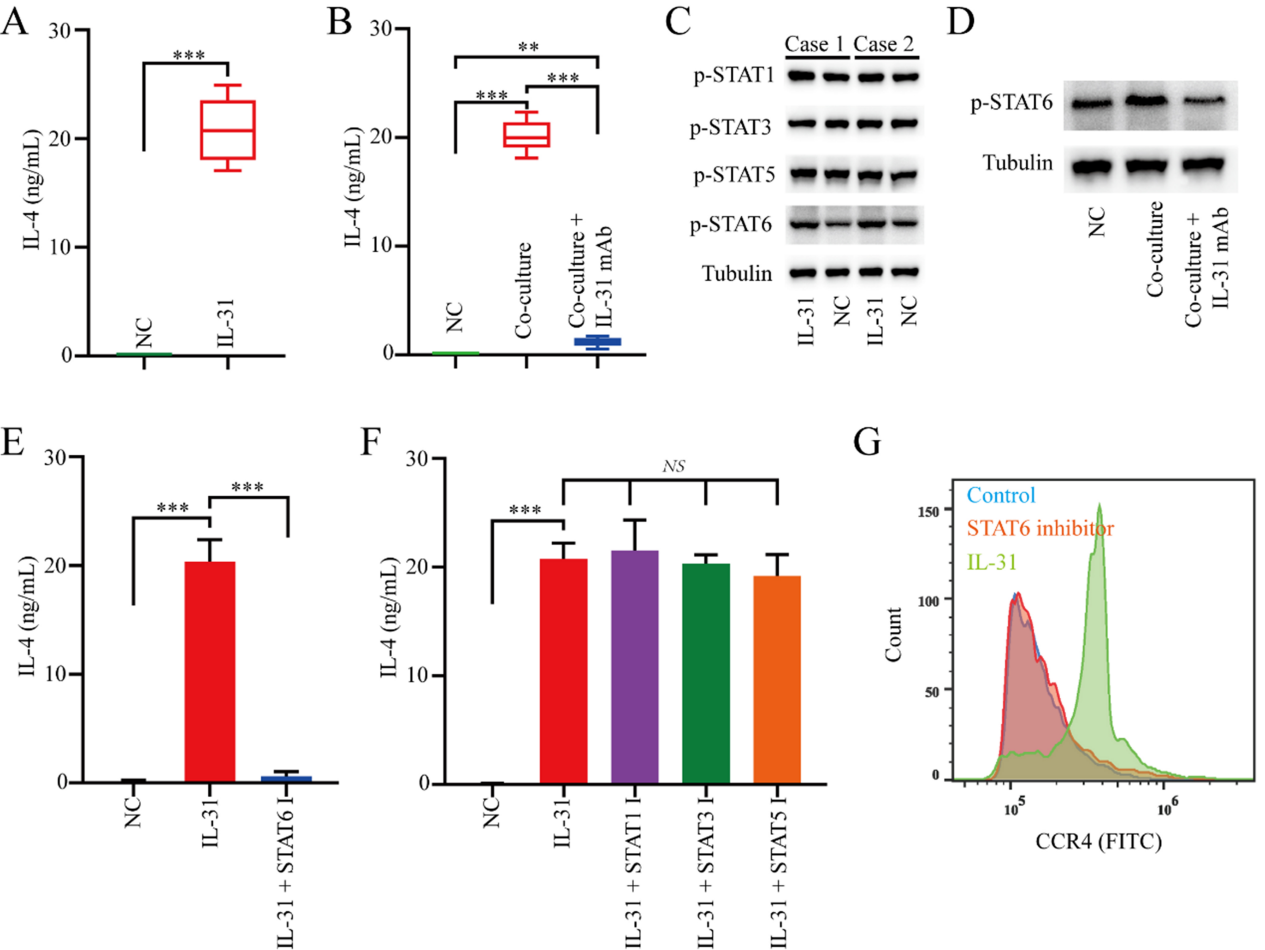
IL-31 induces the polarization of naive CD4^+^ T cells toward Th2 by upregulating the STAT6 signaling pathway. (A) ELISA measuring changes in IL-4 expression in naive CD4^+^ T cells induced by rh-IL-31 (*n* = 3); (B) The increase in IL-4 expression in naive CD4^+^ T cells mediated by FAP^+^CAFs can be blocked by IL-31 antibodies (*n* = 3); (C) Western blot analysis of the effects of rh-IL-31 on signaling pathway activity in naive CD4^+^ T cells; (D) Western blot results showing that the increase in pSTAT6 expression in naive CD4^+^ T cells mediated by FAP^+^CAFs can be blocked by IL-31 antibodies; (E and F) ELISA measuring the effects of relevant small molecule inhibitors on the increase in IL-4 expression in naive CD4^+^ T cells mediated by IL-31 (*n* = 3); (G) Flow cytometric analysis of whether the increase in CCR4 expression in naive CD4^+^ T cells mediated by IL-31 can be blocked by pSTAT6 inhibitor. ^**^P < 0.01; ^***^P < 0.001; bars: means ± standard deviation. CAFs: Cancer-associated fibroblasts.

To elucidate the molecular mechanism of IL-31 in promoting naive CD4^+^ T cells toward Th2 polarization, we subsequently stimulated naive CD4^+^ T cells from healthy donors with rh-IL-31. The results showed that rh-IL-31 significantly upregulated IL-4 levels in naive CD4^+^ T cells [Figure 3A]. Similarly, healthy donors-derived naive CD4^+^ T cells co-cultured with FAP^+^CAFs demonstrated increased protein levels of IL-4 that can be blocked via an IL-31 antibody [Figure 3B].

Subsequently, we explored the signaling pathways by which rh-IL-31 exerts its biological function on naive CD4^+^ T cells. Western blot analysis revealed that rh-IL-31 upregulated the levels of p-STAT6 in naive CD4^+^ T cells [Figure 3C]. Cultured with rh-IL-31 also forced the expression of p-STAT1, p-STAT3, and p-STAT5 in naive CD4^+^ T cells, but to a lesser degree than pSTAT6 [Figure 3C]. Similarly, healthy donors-derived naive CD4^+^ T cells co-cultured with FAP^+^CAFs demonstrated increased protein levels of pSTAT6 can be blocked via IL-31 antibody [Figure 3D].

In addition, the p-STAT6 inhibitor can significantly suppress the increase in IL-4 expression mediated by IL-31 in naive CD4^+^ T cells, while the p-STAT1, p-STAT3, and p-STAT5 inhibitors have no significant effect on the increase in IL-4 expression in naive CD4^+^ T cells mediated by IL-31 [Figure 3E and F]. Importantly, the p-STAT6 inhibitor effectively blocked the IL-31-mediated polarization of naive CD4^+^ T cells into Th2 cells [Figure 3G]. Overall, the findings suggest that IL-31 mediates its biological effects on naive CD4^+^ T cells primarily through p-STAT6 signaling.

### Serum IL-31 levels correlate with anti-PD-1 resistance and Th2 polarization in gastric cancer tissues

**Figure 4:**
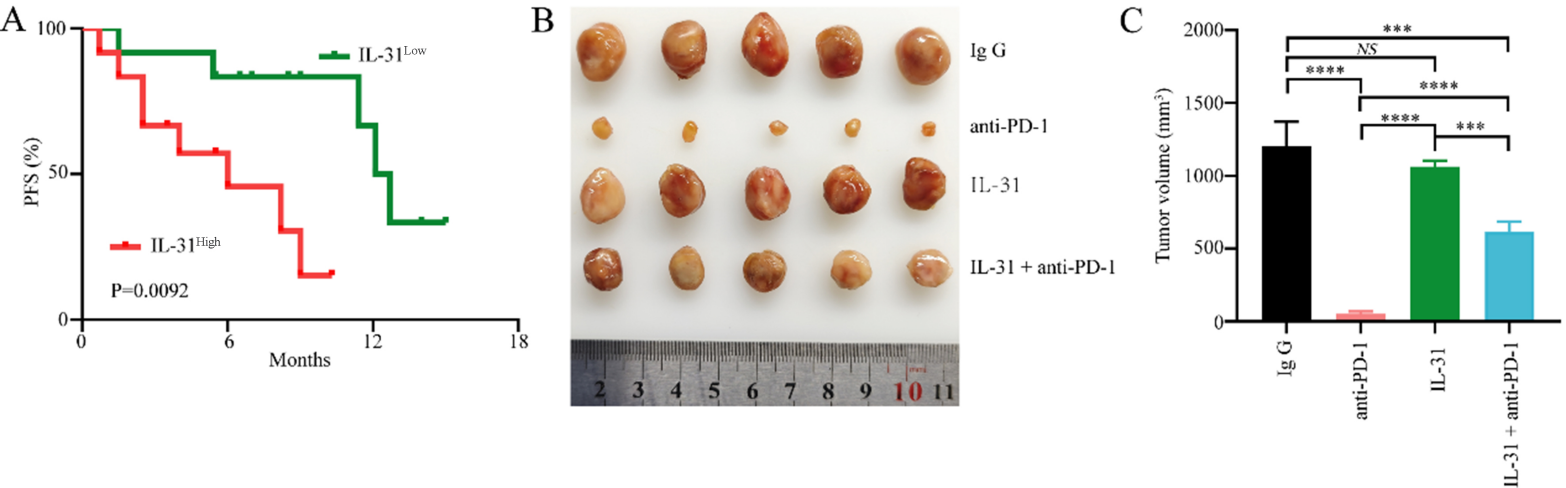
Elevated serum IL-31 levels are associated with resistance to anti-PD-1 in GC. (A) ELISA measuring IL-31 expression levels in the peripheral blood of 24 gastric cancer patients receiving anti-PD-1 treatment, with Kaplan-Meier analysis showing differences in PFS between high and low IL-31 expression groups; (B and C) Establishment of gastric cancer mouse models using MFC cells, examining the relationship between high IL-31 expression and sensitivity of gastric cancer to anti-PD-1 treatment (*n* = 5). ^***^P < 0.001; ^****^P < 0.0001; NS: no significance; bars: means ± standard deviation. GC: Gastric cancer; PFS: progression-free survival.

To further determine the role of FAP^+^CAFs in gastric cancer resistance to anti-PD-1, we analyzed retrospective data from another 24 primary gastric cancer patients receiving combined anti-PD-1 immunotherapy who had not received antitumor therapy [Supplementary Table 3]. Patient demographics and progression-free survival (PFS) were recorded. The expression level of IL-31 in serum was measured by ELISA, while the abundance of FAP^+^CAFs in tumor tissues was assessed via IHC. Kaplan-Meier analysis indicated that patients with high IL-31 levels had significantly shorter PFS compared to those with lower IL-31 levels [Figure 4A]. The median PFS was 6.0 months in the IL-31-high group and 12.4 months in the IL-31-low group. To further evaluate the potential role of a FAP^+^CAFs-mediated IL-31 overexpression in gastric cancer resistance to anti-PD-1 immunotherapy, a tumor model was established by subcutaneously implanting 615 mice with mouse gastric cancer MFC cells. The results showed that IL-31 significantly reduced the therapeutic effectiveness of PD-1 blockade in MFC-derived gastric cancer xenografts [Figure 4B and C]. In addition, to determine whether IL-31 directly promotes gastric cancer cell proliferation, we further incubated MFC cells with recombinant murine IL-31 and assessed its effect using the CCK-8 assay. The results demonstrated that IL-31 had no significant impact on MFC cell proliferation [Supplementary Figure 1].

### FAP^+^CAFs may influence CD8^+^ T cell function through the polarization of naive CD4^+^ T cells toward Th2

**Figure 5:**
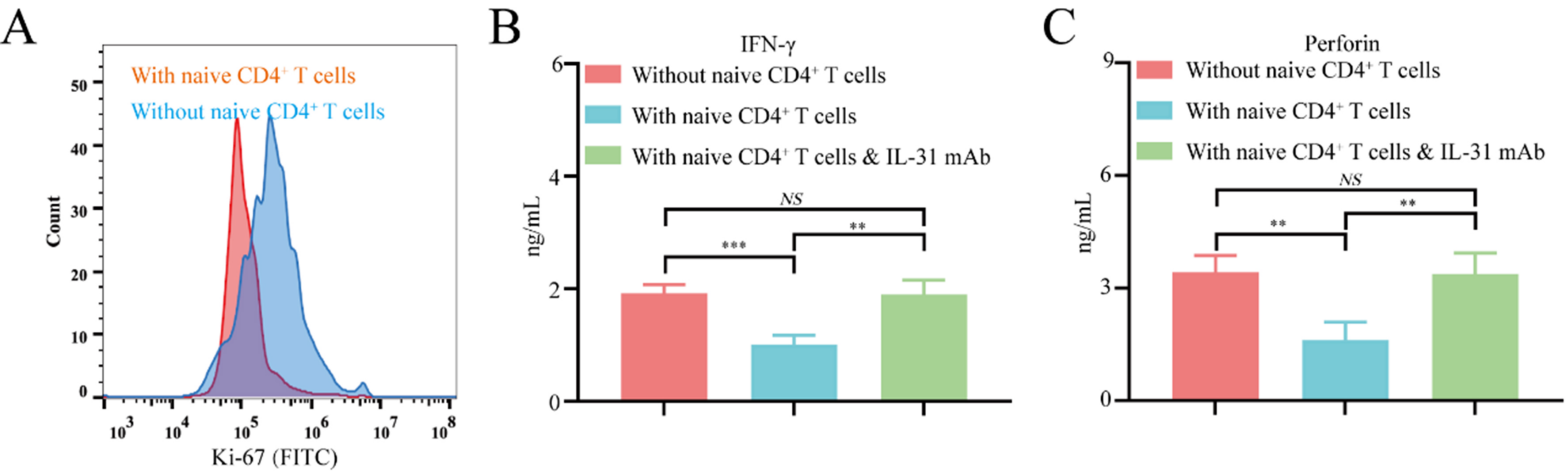
FAP^+^CAFs may inhibit CD8^+^ T cell function by promoting the polarization of naive CD4^+^ T cells toward Th2. (A) Flow cytometric analysis of the effect of FAP^+^CAFs on CD8^+^ T cell proliferation with or without the presence of naive CD4^+^ T cells. (B and C) ELISA measuring whether IL-31 antibodies can block the decrease in IFN-γ and perforin expression in CD8^+^ T cells mediated by co-culture with FAP^+^CAFs and CD4^+^ T cells (*n* = 3). ^**^P < 0.01; ^***^P < 0.001; NS: no significance; bars: means ± standard deviation. CAFs: Cancer-associated fibroblasts.

To further elucidate the mechanism by which FAP^+^CAFs and IL-31 mediate the polarization of naive CD4^+^ T cells into Th2 cells during immune evasion in gastric cancer, CD8^+^ T cells isolated from healthy donor peripheral blood mononuclear cells (PBMCs) were co-cultured either with FAP^+^CAFs alone or with both FAP^+^CAFs and naive CD4^+^ T cells. When co-cultured with FAP^+^CAFs and naive CD4^+^ T cells for 72 h, CD8^+^ T lymphocytes showed a significantly inhibited proliferation ability compared to co-cultured with FAP^+^CAFs alone [Supplementary Figure 2 and Figure 5A]. In addition, co-cultured with FAP^+^CAFs and naive CD4^+^ T cells for 72 h, CD8^+^ T lymphocytes showed a significantly decreased IFN-γ and perforin secretion compared to co-cultured with FAP^+^CAFs alone [Figure 5B and C]. In addition, the reduction in IFN-γ and perforin expression in CD8^+^ T cells induced by a naive CD4^+^ T cell co-culture system can be blocked by IL-31 blocking antibodies [Figure 5B and C]. These results indicate that FAP^+^CAFs might inhibit the proliferation of CD8^+^ T cells and contribute to their functional exhaustion by promoting the polarization of naive CD4^+^ T cells toward Th2.

### FAP^+^CAFs are associated with an immunosuppressive TME and reduced sensitivity to anti-PD-1 therapy in gastric cancer

**Figure 6:**
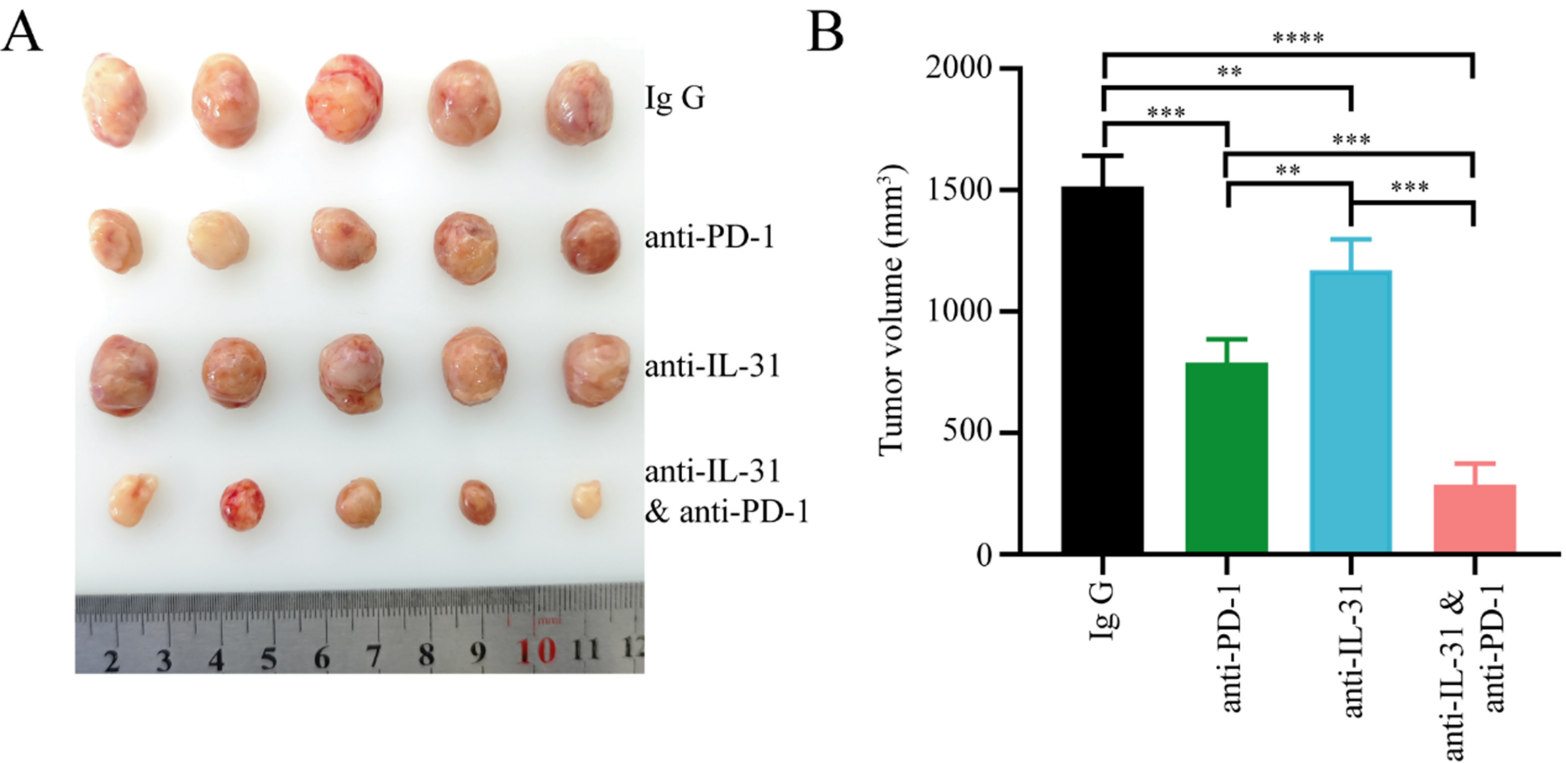
IL-31 blockade enhances anti-PD-1 therapy in gastric cancer, potentially counteracting the immunosuppressive influence of FAP^+^CAFs. (A and B) CAFs were isolated from gastric cancer tissues, and FAP expression in CAF cells was upregulated using lentiviral vectors. FAP^+^CAFs were mixed with gastric cancer cell lines and implanted into humanized mice. Treatment began when the tumor volume reached approximately 50 mm^3^. The results showed that tumors treated with anti-PD-1 or anti-IL-31 monotherapy showed moderately reduced volumes compared to controls, while the combination of anti-PD-1 and anti-IL-31 antibodies led to a significantly greater reduction in tumor size (*n* = 5). ^**^P < 0.01; ^***^P < 0.001; ^****^P < 0.0001; bars: means ± standard deviation. CAFs: Cancer-associated fibroblasts.

To further evaluate whether FAP^+^CAFs contribute to remodeling the tumor immune microenvironment and reducing the efficacy of anti-PD-1 therapy in gastric cancer, we isolated CAFs from gastric cancer tissues and upregulated FAP expression in CAFs cells through lentiviral vectors, establishing a cell line of FAP overexpressing CAFs derived from gastric cancer patients (FAP^+^CAFs) [Figure 2D]. FAP^+^CAFs were mixed with gastric cancer cell lines and implanted into humanized mice. When the tumor volume reached approximately 50 mm^3^, treatment with PD-1 monoclonal antibody was initiated. The results showed that FAP^+^CAFs may impair the efficacy of anti-PD-1 therapy in gastric cancer mice, and that IL-31 blockade can partially restore treatment sensitivity, potentially by alleviating the immunosuppressive effects associated with FAP^+^CAFs [Figure 6A and B].

## DISCUSSION

In recent years, the heterogeneity of CAFs and their role in tumor immune evasion have gained increasing attention from oncologists^[21-23]^. While several studies have examined the contribution of CAFs to gastric cancer progression and immune suppression^[24]^, the precise molecular mechanisms through which specific CAF subtypes influence immune evasion in gastric cancer remain incompletely understood. Clarifying the heterogeneity of CAFs and the molecular mechanisms underlying the immunosuppressive microenvironment induced by interactions between CAFs and gastric cancer or immune cells is crucial for improving therapeutic outcomes. In this study, we identified significant variations in the expression levels of fibroblast activation protein (FAP) within CAFs across tumor tissues from various gastric cancer patients. Notably, an elevated presence of FAP^+^CAFs was observed in gastric cancer tissues exhibiting resistance to PD-1 monoclonal antibody treatment.

Previous research has demonstrated that CAFs communicate with gastric cancer cells via diverse molecular mechanisms^[24]^. For instance, SULF1 secreted by CAFs binds to TGFBR3 on the surface of gastric cancer cells, alters its interaction with TGF-β1, and subsequently facilitates the activation of the TGF-β signaling pathway, thereby promoting metastasis and resistance to cisplatin^[25]^. IL-8 secreted by CAFs induces the upregulation of PD-L1 in gastric cancer cells through activation of the p38/JNK/NF-κB pathway, facilitating immune evasion^[26]^. However, these studies largely overlook the potential role of specific CAF subtypes in modulating immune escape mechanisms. Our research identified a subset of CAFs derived from gastric cancer patients that exhibit high expression of FAP (FAP^+^CAFs) and demonstrate resistance to anti-PD-1 therapy. Previous study in the tumor tissues of metastatic non-small cell lung cancer (NSCLC) also demonstrated a significant enrichment of FAP^+^CAFs, with their abundance correlating with poor OS^[27]^. Yet, the functional role and mechanisms of FAP^+^CAFs in immune evasion have remained largely unexplored. Our study provides new evidence, revealing that FAP^+^CAFs secrete high levels of IL-31, which plays a critical role in this immune escape process.

T helper (Th) cells are recognized as key players in orchestrating antitumor immune responses^[28]^. The balance between Th1 and Th2 cells, two key subtypes of Th cells, is crucial in determining tumor progression or suppression. Disruption of the Th1/Th2 balance is known to contribute to the development of malignancies^[29]^. In the TME, an imbalance may shift Th0 cells toward Th2 polarization, promoting immune suppression. Th1 cells exert antitumor effects by enhancing CD8^+^ T cell proliferation, upregulating granzyme B, and promoting immune cell cytotoxicity through the secretion of IFN-γ and IL-2. Moreover, Th1 cells activate dendritic cells (DCs) through the production of IFN-γ^[29]^. Conversely, Th2 cells are primarily involved in promoting tumor progression through the recruitment and activation of immunosuppressive cells within TME. Specifically, Th2 cells induce M2 macrophage polarization through the secretion of IL-4 and IL-10^[29]^. Additionally, studies have shown a positive correlation between Th2-derived cytokines and the accumulation of MDSCs in tumor tissues. Our findings further demonstrate that IL-31 derived from FAP^+^CAFs promotes the polarization of naive CD4^+^ T cells toward the Th2 phenotype, thereby establishing an immunosuppressive TME and facilitating gastric cancer immune evasion.

Here, our study uncovers a novel mechanism of CAF-mediated immune evasion in gastric cancer: FAP^+^CAFs facilitate the transition of naive CD4^+^ T cells toward a Th2 phenotype through IL-31 secretion. This process further activates the STAT6 signaling pathway through the IL-31R receptor, thereby reinforcing the immunosuppressive nature of the TME. Our findings expand upon previous studies by elucidating the specific role of FAP^+^CAFs in reshaping the immune landscape of gastric cancer, highlighting their potential as therapeutic targets. Although the primary focus of this study is on the role of IL-31 in modulating response to immune checkpoint therapy, our data also suggest that IL-31 alone does not directly promote tumor growth, as exogenous IL-31 had no significant effect in the murine model without immunotherapy. In contrast, IL-31 blockade resulted in modest tumor suppression in the CAF-containing model, indicating that CAF-derived IL-31 likely exerts its effects by shaping an immunosuppressive microenvironment rather than driving tumor proliferation per se.

Despite the novel insights provided by our study, several limitations should be acknowledged. Most notably, in the *in vivo* model where gastric cancer cells were co-injected with FAP^+^CAFs, we did not include a tumor-only control group without FAP^+^CAFs. Therefore, while anti-PD-1 treatment showed partial tumor inhibition, the absence of a FAP^+^CAF-free group limits our ability to definitively attribute reduced immunotherapy efficacy to FAP^+^CAF-mediated resistance. In addition, our mechanistic investigations focused primarily on the FAP^+^CAF–IL-31 axis, and the potential contributions of other CAF subtypes or cytokines remain to be explored. Lastly, while we propose IL-31–STAT6 signaling as a central mechanism, other downstream pathways might also be involved and warrant future exploration.

In summary, our results reveal a novel mechanism by which IL-31 secreted by FAP^+^CAFs drives immune evasion in gastric cancer. Targeting the IL-31-driven transition of naive CD4^+^ T cells toward a Th2 phenotype provides a potential approach to improve the efficacy of immunotherapy. Additional studies are warranted to elucidate the molecular mechanisms underlying the different subtypes of CAFs and the roles of other CAF subtypes in gastric cancer immunity.

## DECLARATIONS

### Availability of data and materials

The original contributions presented in the study are included in the article/Supplementary Materials; further inquiries can be directed to the corresponding authors.

### Copyright

© The Author(s) 2025
