# Supplementary material for: Effects of FAP+ cancer-associated fibroblasts on anti-PD-1 immunotherapy and CD4+ T cell polarization in gastric cancer
Source: Cancer Drug Resist. 2025 Jul 29;8:38. doi: 10.20517/cdr.2025.97 (PMC12366491; doi:10.20517/cdr.2025.97)
Supplement: Supplementary file 1 [file cdr-8-38-SupplementaryMaterials.pdf]

Supplementary Materials

Effects of FAP$^+$ cancer-associated fibroblasts on anti-PD-1 immunotherapy and CD4$^+$ T cell polarization in gastric cancer

Jing Wu[1,2,3,#], Peng-Fei Zhang[2,3,#], Yu Zeng[4,#], Ya-Nan Hai[5,#], Kun-Ming Zhang[6], Shu Dong[7], Ji-Chong Xu[8], Lan-Lin Zhang[1], Zhi-Xiong Wu[1], Hong Jiang[1]

[1]Department of Cancer Center, Tongji Hospital, Tongji University School of Medicine, Shanghai 200065, China.

[2]Department of Medical Oncology, Zhongshan Hospital, Fudan University, Shanghai 200032, China.

[3]Department of Medical Oncology, Shanghai Geriatric Medical Center, Shanghai 201104, China.

[4]Department of Pathology, Tongji Hospital, Tongji University School of Medicine, Shanghai 200065, China.

[5]Department of Oncology, Shanghai East Hospital, Tongji University School of Medicine, Shanghai, 200120, China.

[6]Department of Oncology, People's Hospital of Longhua, Shenzhen 518109, Guangdong, China.

[7]Department of Integrative Oncology, Fudan University Shanghai Cancer Center, Shanghai 200032, China.

[8]Department of Interventional Radiology, Tongji Hospital, Tongji University School of Medicine, Shanghai 200065, China.

[#]Authors contributed equally.

Correspondence to: Prof. Hong Jiang, Department of Cancer Center, Tongji Hospital, Tongji University School of Medicine, No. 389 Xincun Road, Putuo District, Shanghai 200065, China. E-mail: Jianghong09123@126.com

Supplementary Experimental Methods

CAFs isolation

CAFs isolation was followed the method previously reference (J Clin Invest. 2023; 133(5): e147087.). In brief, fresh gastric cancer tissues were processed within 30 minutes after surgical resection and washed three times with PBS containing 1% penicillin-streptomycin. The gastric cancer tissues were then minced and digested with papain at 37°C for 30 minutes, followed by vortex mixing to obtain a homogeneous suspension. Subsequently, the suspension was filtered through a 70 μm cell strainer and rinsed with culture medium containing 1% penicillin-streptomycin. After centrifugation and red blood cell lysis, the cell pellet was resuspended in DMEM/F12 medium supplemented with 10% fetal bovine serum (FBS) and 1% penicillin-streptomycin. Cancer-associated fibroblasts (CAFs) were isolated using differential adhesion time, followed by multiple rounds of sequential digestion with 0.25% trypsin-EDTA. After approximately 5 weeks of culture (around 5 passages), a morphologically fibroblast-like cell population was finally obtained.

Co-culture Model of FAP^+ CAFs, $CD8^+$ T Cells, and Naïve $CD4^+$ T Cells

In this co-culture system, FAP^+CAFs were plated at 5×10^5 cells/well in 6-well plates. $CD8^+$ T cells (1×10^6 cells/well) and naïve $CD4^+$ T cells (1×10^6 cells/well) were separately seeded in Millicell® hanging cell culture inserts. Two experimental groups were established: (1) FAP^+CAFs co-cultured with $CD8^+$ T cells, and (2) FAP^+ CAFs co-cultured with both $CD8^+$ T cells and naïve $CD4^+$ T cells. After 72 hours of co-culture, $CD8^+$ T cell proliferation was quantified by flow cytometry.

Isolation of $CD8^+$ T Cells

$CD8^+$ T cells were isolated using the EasySep™ Direct Human $CD8^+$ T Cell Isolation Kit (Catalog#19663) according to the manufacturer's protocol.

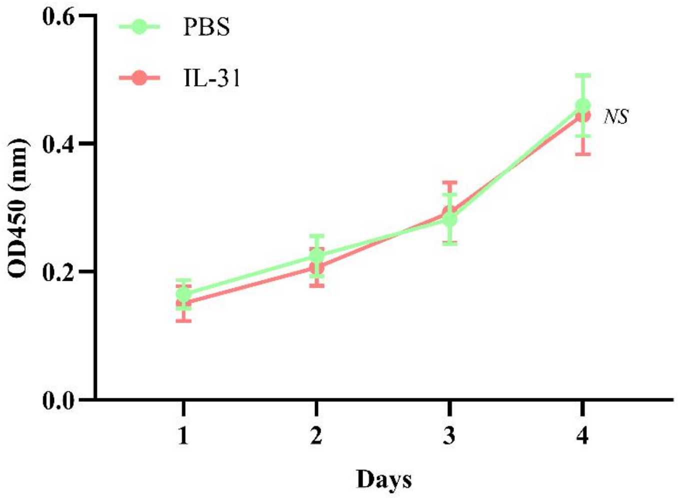

Supplementary Figure 1. MFC cells were seeded in 96-well plates at a density of 200 cells per well. After adherence, the cells were cultured in medium containing either recombinant murine IL-31 (10 ng/mL) or vehicle control (PBS). Proliferation of MFC cells was assessed using the CCK-8 assay. The results indicated that IL-31 had no significant effect on the proliferative capacity of murine gastric cancer cells.

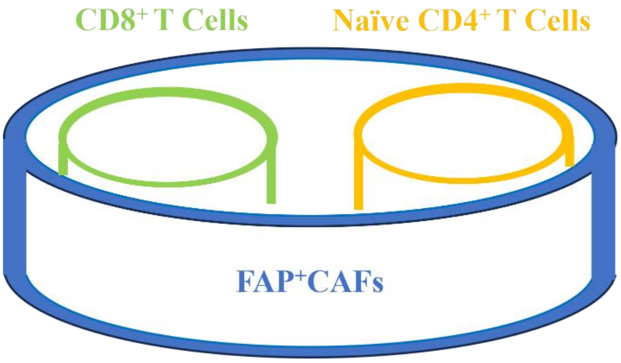

Supplementary Figure 2. Co-culture Model of FAP$^+$ CAFs, CD8$^+$ T Cells, and Naïve CD4$^+$ T Cells.

Supplementary Table 1. The clinical characteristics of 20 gastric cancer patients

Variables	Number (%)
Age (years)	
<60	12 (60%)
≥60	8 (40%)
Gender	
Male	14 (70%)
Female	6 (30%)
Liver metastasis	
No	3 (15%)
Yes	17 (85%)
Lymph node metastasis	
No	1 (5%)
Yes	19 (95%)
Degree of differentiation	
Well	7 (35%)
Poor	13(65%)
Lauren classification	
Intestinal type	4 (20%)
Diffuse and Mixed types	16 (80%)
TNM staging	
I–III	0 (0)
IV	20 (100%)

Supplementary Table 2. Prognosis value and positive association of FAP^+CAFs in 100 gastric cancer patients

Variables	FAP^+CAFs		
	**High**	**Low**	***P* value**
Age (years)			
<60	31	27	0.544
≥60	19	23	
Gender			
Male	35	33	0.83
Female	15	17	
Lymph node metastasis			
No	1	5	0.204
Yes	49	45	
Degree of differentiation			
Well	21	24	0.688
Poor	29	26	
Lauren classification			
Intestinal type	7	11	0.436
Diffuse and Mixed types	43	39	

Supplementary Table 3. The clinical characteristics of 24 gastric cancer patients

**Variables**	**Number(%)**
Age (years)	
<60	9 (37.5%)
≥60	15(62.5%)
Gender	
Male	17 (70.8%)
Female	7(29.2%)
Liver metastasis	
No	0 (0)
Yes	24 (100%)
Lymph node metastasis	
No	2 (8.3%)
Yes	22 (91.7%)
Degree of differentiation	
Well	8 (33.3%)
Poor	16 (66.7%)
Lauren classification	
Intestinal type	3 (12.5%)
Diffuse and Mixed types	21(87.5%)
TNM staging	
I–III	0 (0)
IV	24 (100%)
